# The impact of temporal fine structure and signal envelope on auditory motion perception

**DOI:** 10.1371/journal.pone.0238125

**Published:** 2020-08-21

**Authors:** Michaela Warnecke, Z. Ellen Peng, Ruth Y. Litovsky

**Affiliations:** University of Wisconsin-Madison, Waisman Center, Madison, WI, United States of America; Medical University Hannover; Cluster of Excellence Hearing4all, GERMANY

## Abstract

The majority of psychoacoustic research investigating sound localization has utilized stationary sources, yet most naturally occurring sounds are in motion, either because the sound source itself moves, or the listener does. In normal hearing (NH) listeners, previous research showed the extent to which sound duration and velocity impact the ability of listeners to detect sound movement. By contrast, little is known about how listeners with hearing impairments perceive moving sounds; the only study to date comparing the performance of NH and bilateral cochlear implant (BiCI) listeners has demonstrated significantly poorer performance on motion detection tasks in BiCI listeners. Cochlear implants, auditory protheses offered to profoundly deaf individuals for access to spoken language, retain the signal envelope (ENV), while discarding temporal fine structure (TFS) of the original acoustic input. As a result, BiCI users do not have access to low-frequency TFS cues, which have previously been shown to be crucial for sound localization in NH listeners. Instead, BiCI listeners seem to rely on ENV cues for sound localization, especially level cues. Given that NH and BiCI listeners differentially utilize ENV and TFS information, the present study aimed to investigate the usefulness of these cues for auditory motion perception. We created acoustic chimaera stimuli, which allowed us to test the relative contributions of ENV and TFS to auditory motion perception. Stimuli were either moving or stationary, presented to NH listeners in free field. The task was to track the perceived sound location. We found that removing low-frequency TFS reduces sensitivity to sound motion, and fluctuating speech envelopes strongly biased the judgment of sounds to be stationary. Our findings yield a possible explanation as to why BiCI users struggle to identify sound motion, and provide a first account of cues important to the functional aspect of auditory motion perception.

## Introduction

Cochlear implants (CIs) are auditory prostheses offered to individuals with profound hearing loss. The devices were originally designed to provide patients with access auditory input for oral communication [1,2]. Bilateral implantation is known to provide access to spatial hearing cues (e.g. [3,4]); these are important for localizing sound sources, which is vital to successful navigation in complex auditory environments. Previous research has shown that bilateral cochlear implant (BiCI) users generally do not achieve the same spatial hearing accuracy as their normal-hearing (NH) peers in sound localization tasks [3–6]. This disadvantage for BiCI users may be partly explained by the signal processing strategy of cochlear implants, which encodes the signal’s temporal envelope (ENV) and removes the temporal fine structure (TFS) [2,7]. NH listeners have access to interaural timing differences (ITDs) in the TFS, which are more useful than ITDs in the ENV and likely to facilitate accurate sound localization [8–11]. By contrast, BiCI users only have access to interaural level differences (ILDs) and ITDs in the signal ENV to lateralize and localize sounds in space (e.g. [6,12–16]), which puts them at a potential disadvantage compared to NH listeners.

While stationary sound localization has been the focus of psychoacoustic research in recent decades [8,17], sound motion is a common aspect of the auditory environments in natural everyday situations, either because sound sources move or the listener is in motion. Recent work showed that BiCI users have difficulty distinguishing between stationary and moving sounds as compared to NH listeners [18]. Given that both TFS and signal ENV cues are available to NH listeners, while only ENV cues are available to BiCI listeners, this study aims to understand the usefulness of TFS and signal ENV in auditory motion perception.

In human psychoacoustics, sensitivity to moving sounds is difficult to quantify. Unlike stationary sounds, moving sounds traveling along a particular trajectory are subject to dynamically changing parameters such as stimulus velocity and duration, which co-vary in a way that each may provide useful auditory cues for motion perception [19–23]. Several studies have investigated the smallest angular distance a sound has to travel for its movement direction to be correctly identified, which is known as the minimal audible movement angle (MAMA) (for a recent review, see [23]). Across studies measuring the MAMA, a variety of stimuli have been used, covering different spectral bandwidths, playback durations, and moving velocities. For NH adults, the MAMA ranges between 2° and 10° for sounds moving along the horizontal plane [23,24].

The research into the MAMA has almost exclusively focused on NH listeners, utilizing stimuli which are largely uncharacteristic of naturally occurring sounds to explore the underlying mechanism of motion perception in the healthy auditory system. For listeners with hearing loss who utilize BiCIs, auditory motion perception is an equivalently critical functional ability that facilitates navigation of everyday auditory environments. It has been reported that the MAMA is approximately 20° for older adults using bilateral hearing aids [24]; auditory motion is largely unexplored among listeners with BiCIs. Recently, Moua and colleagues investigated how parameters such as stimulus duration and velocity impacted the ability of NH and BiCI listeners to detect sound motion [18]. The results show that the NH group outperformed the BiCI group on several measures of auditory motion, including better accuracy in identifying sound motion, discriminating movement direction, and determining angular displacement.

It remains unclear why BiCI users struggle to detect sound motion. It is conceivable that CI processing may deprive BiCI users of acoustic cues that could be useful in the detection of moving sounds. Briefly, in CI processing, the broadband acoustic signal is bandpass-filtered into several “spectral channels.” For each channel, the temporal signal ENV is extracted and it subsequently amplitude-modulates constant pulse trains that have a frequency of approximately 1000 Hz [2,7]. This high-rate pulse train preserves the temporal ENV in each spectral channel, which is important for the ability of CI users to achieve high levels of speech perception in quiet [3,25]. However, by discarding the original TFS of the signal and replacing it with a high-frequency pulse train carrier [2,7,26], CI processing renders binaural cues for localization spurious and poor [16,25,27]. For NH listeners, earlier work has illustrated that TFS ITD, conveyed through low-frequency acoustic signals at < 2.5 kHz, governs sound lateralization [28,29] and strongly affects localization of stationary sounds [30–33]. NH listeners do not seem to consistently rely on signal ENV for sound localization, especially at lower frequencies [11,13,28,29].

When considering the role of ENV and TFS cues in sound localization, previous research has shown that BiCI users (who have access to signal ENV, but not TFS) localize speech signals more accurately than noise signals [13], though the underlying reason for this is unclear. No such effect has been found for NH listeners [13]. By studying NH listeners’ sound motion perception for acoustic cues that are unavailable to BiCI listeners, but not to NH listeners, we can begin to understand each cues’ relevance to sound motion perception and processing strategies for CIs.

To test the relative impacts of signal TFS and ENV on auditory motion perception, we implemented a modified version of Smith and colleagues’ ([28]) approach to generate acoustic chimaera signals. A chimaera is an acoustic stimulus resulting from the artificial merging of the ENV and TFS information of two separate sounds (see Fig 2 in [28]). For the purposes of our study, we created chimaeras from the ENV and TFS information of speech and spectrally-matched noise (SMN), by merging the speech ENV with the SMN TFS, and vice versa. We thus assessed the relative contributions of TFS and ENV on correct identification of sound motion and response bias. As low-frequency TFS provides important ITD cues for stationary sound localization, we hypothesized that NH listeners’ abilities to identify sound motion would be reduced with chimaera stimuli that are high-pass (HP) filtered to remove low-frequency TFS as compared to broadband chimaeras. In addition, a speech ENV contains stronger fluctuations than a flat noise ENV, thus creating more opportunity for availability of interaural difference cues. As such, we expected that NH listener’s abilities to detect sound motion would be improved for chimaera stimuli with speech ENVs, as compared to chimaeras with noise ENVs.

## Materials and methods

### Participants

Nine listeners (ages 19 to 32 years, avg. 22 years) participated in this study, and received either university credit or payment for their participation. All listeners passed hearing screening at octave frequencies between 250 and 8000 Hz, defined as thresholds ≤ 20 dB, and none had extensive experience as research participants in psychoacoustic studies. All participants were naïve to the study’s experimental design and purpose, and gave written informed consent prior to experiment. All experimental procedures followed the regulations set by the National Institutes of Health and were approved by the University of Wisconsin’s Health Sciences Institutional Review Board.

### Test stimuli and experimental design

In the two main experiments described below, speech tokens were 112 unique disyllabic words from the TVM corpus [34], spoken by multiple male and female talkers and recorded at 44.1 kHz. All words started with a consonant. The collection of words had an average duration of 519 ms, ranging from 401 to 600 ms. Fifty-six words were used for Experiment I and the remaining 56 words were used for Experiment II without repetition.

To create variations of individual speech tokens, a matching SMN was created for each word by synthesizing noise of the same power spectrum and duration via randomizing the phase of its fourier spectrum. To create chimaeras of the speech and SMN signals, we utilized Smith et al.’s Chimaera-generating approach [28]. The speech and SMN stimuli were each bandpass-filtered into eight frequency bands between 80 to 8000 Hz with equal bandwidth [35]. The cutoff frequencies for the eight channels were 200 Hz, 391 Hz, 694 Hz, 1172 Hz, 1927 Hz, 3122 Hz, and 5012 Hz. Subsequently, for each frequency band, the ENV and TFS of both the speech and SMN signal were extracted using Hilbert transform and exchanged, such that the ENV of one signal was superimposed on the TFS of the other, and vice versa. The eight-band signals were summed in the time domain to form a chimaera. For each pair of a speech token and its matching SMN, two chimaeras were created, one containing speech ENV and the other containing SMN ENV. For the remainder of this study, speech chimaera (SC) refers to stimuli with a speech ENV and SMN TFS, whereas noise chimaera (NC) refers to stimuli with a SMN ENV and speech TFS (see Table 1). To investigate the effect of low-frequency TFS, another set of chimaera stimuli was created by high-pass filtering each frequency band’s extracted TFS component at 2.5 kHz using a 6^th^-order Butterworth filter. This process ensured the preservation of the envelope, while effectively removing low-frequency TFS content, and produced a HP-filtered speech (SC_hp) and noise chimaera (NC_hp). A total of 672 stimuli were created from the six versions of stimuli for each token in the 112 disyllabic words. Table 1 provides a summary of the stimuli: 1. Original speech (OS); 2. Spectrally-matched noise (SMN); 3. Speech chimaera (SC); 4. Noise chimaera (NC); 5. HP speech chimaera (SC_hp); 6. HP noise chimaera (NC_hp). These six stimulus conditions fall into one of three categories: Unprocessed stimuli (OS, SMN), Chimaera stimuli (SC, NC), and Chimaera HP stimuli (SC_hp, NC_hp). Note that each stimulus condition contains either speech (OS, NC, NC_hp) or SMN (SMN, SC, SC_hp) in the TFS, and has either a fluctuating speech (OS, SC, SC_hp) or a flat noise (SMN, NC, NC_hp) envelope. Previous work evaluating horizontal localization of speech and noise stimuli found that the lateral angle away from the median plane was estimated accurately for both types of stimuli [36,37]. To ensure that the signal ENV was retained well in the chimaera stimuli so that participants could distinguish between speech and noise, we assessed speech understanding (see below).

**Table 1:** Experimental conditions and data.

				Experiment I		Experiment II		
Category	Condition	ENV	TFS	Sensitivity (mean ± sem)	Response bias (mean ± s.e.m)	Sensitivity (mean ± sem)	Response bias (mean ± sem)	Speech understanding (mean ± sem)
Unprocessed	OS	Fluctuating	Speech	0.59 ± 0.23	-0.54 ± 0.32	2.37 ± 0.23	-0.31 ± 0.19	99.8% ± 0.2%
	SMN	Flat	SMN	2.02 ± 0.40	0.42 ± 0.06	3.38 ± 0.32	0.38 ± 0.16	0% ± 0%
Chimaera	SC	Fluctuating	SMN	1.25 ± 0.21	-0.44 ± 0.30	2.54 ± 0.28	0.14 ± 0.23	90.1% ± 1.3%
	NC	Flat	Speech	1.32 ± 0.27	0.53 ± 0.18	2.48 ± 0.39	0.89 ± 0.17	43.9% ± 2.7%
Chimaera HP	SC_hp	Fluctuating	SMN HP	0.51 ± 0.18	-0.43 ± 0.30	1.26 ± 0.14	-0.27 ± 0.31	12.0% ± 2.3%
	NC_hp	Flat	Speech HP	0.48 ± 0.13	0.26 ± 0.19	1.52 ± 0.24	0.42 ± 0.30	0.1% ± 0.1%

Table describes experimental parameters (Category, Condition) and their respective envelope (ENV) and temporal fine structure (TFS) contents. Sensitivity, response bias and speech perception scores are provided for each condition as mean **±** standard error of the mean (sem).

Prior to the main experiments, we tested participants’ stationary localization using the same six types of stimuli (Table 1). Stationary sound localization accuracy was measured using 78 stimuli created from 13 monosyllabic words with an averaged duration of 513 ms (ranging from 450 to 547 ms). Stationary localization was tested at 13 different speaker locations (-60°, -50°, - 40°, -30°, -20°, -10°, 0°, + 10°, + 20°, + 30°, + 40°, + 50° and + 60°), and participants responded by indicating the location of the sound without having to recognize the word. Hence, speech understanding was not collected. The stationary sound localization served to familiarize participants with the testing procedure, the chimaera stimuli, and tested the effect of low-frequency TFS and signal ENV on stationary localization. We calculated root mean square (RMS) errors as a measure of localization error per stimulus condition.

During the main experiments, there were seven onset locations along the horizontal loudspeaker array (- 60°, - 40°, - 20°, 0°, + 20°, + 40°, and + 60°). For each onset location, and each condition (see above), there were 8 trials [4 stationary trials, 4 moving trials (two leftward and two rightward motion)], totaling 672 trials (7 locations x 4 stationary trials x 4 moving trials x 6 conditions) per participant. All stimuli were pseudo-randomly assigned to play back at each onset location. All moving sounds traveled a 10° angular distance in Experiment I and 20° angular distance in Experiment II. The moving sounds always ended inside the ± 60° azimuthal range. To minimize a learning effect from the experimental order and potential priming of auditory motion after effects [38,39], all stimuli, playback locations and motion types were pseudo-randomized. Additionally, the order for completing Experiments I and II was randomized amongst participants. We calculated sensitivity and response bias as a measure of sound motion identification.

### Apparatus

All testing was done in a sound booth (internal dimensions: 2.9 m x 2.74 m x 2.44 m; Acoustic Systems, Austin, TX, USA) covered in acoustic foam on the walls and ceiling (Pinta Acoustics, Minneapolis, MN, USA). Participants sat in a chair in the middle of a horizontal 37-loudspeaker array (Cambridge SoundWorks, North Andover, MA, USA; TDT Technologies, Alachua, FL, USA), which spanned azimuthal locations from—90° (left) to + 90° (right) in 5° resolution. The speaker array was hidden behind a black curtain that was acoustically transparent to remove visual cues from the speakers. The vector-base amplitude panning (VBAP) algorithm was implemented to create a continuously moving sound source along a trajectory, by panning between groups of two adjoining loudspeakers [18,40]. Slight deviations in the frequency responses of individual loudspeakers may cause spectro-temporal variations that could provide a cue for motion detection when sounds are panned between two adjoining speakers [41]. To control for such potential bias, we implemented time-domain inverse filters for each loudspeaker to correct for flat frequency responses at the loudspeaker output. Individual loudspeakers were calibrated to have the same output intensity to ensure smooth panning. At each location, the stationary sounds were calibrated to be 65 dBA SPL at the location of the participant’s head using a sound level meter (System 824, Larson Davis, Depew, NY, USA). For all moving sounds, the gain coefficients for motion panning between the two adjoining speakers together were adjusted to an output level normalized to 65 dBA.

Four infrared cameras (OptiTrack, Natural Point Inc., Corvallis, OR, USA) were mounted to the ceiling of the sound booth and continuously monitored reflective markers attached to a small, custom-built hand-held laser pointer, which the participant used to indicate the location of the perceived sound. A small touchscreen (34 cm, 13.3 inch diagonal; OnLap 1303, GeChic, Taichung City 403, Taiwan) was provided to the participant to start a trial and provide additional responses during the experiment.

### Procedure

The same dual-task was used for both Experiment I and II. For each trial, listeners were asked to identify both auditory motion (i.e., track stationary location vs. moving trajectory) and the word token. On a given trial, participants pressed the touchscreen to start the trial. Subsequently, a sound (moving or stationary) played at a location along the horizontal speaker array. Participants were instructed to face forward and keep their head still during sound presentation; no physical movement restrictions were enforced. After sound offset, participants could move their head. They were instructed to use the laser pointer to indicate the perceived sound location (for stationary sounds) or trace the sound from its perceived onset to offset locations (for moving sounds). After indicating perceived sound location/tracking, the frontal touch screen displayed a matrix with words from the experiment. To indicate the perceived word token, participants could either select a word from the word matrix, or a button to indicate that they did not recognize the word. After submitting an answer, participants could start a new trial by pressing the touch screen. No feedback on sound motion or the acoustic stimulus was given. In order to stay consistent with the response mechanism employed during stationary localization testing, we asked participants to trace trajectories of moving sounds using the laser pointer, rather than utilizing a 2-alternative forced choice (2-AFC) task to indicate whether a sound moved.

### Data analysis

We were interested in listeners’ sensitivity in detecting sound motion, but not precision in tracking the trajectory of the moving sound. Motion tracking data were collected as angular displacement. Subsequently, we categorized the tracked angular displacements into “stationary” and “moving” trials using *k-means* clustering (k = 2; S1 Fig). This conversion allowed us to treat these data as categorical measures of sound motion detection (i.e., stationary/moving).

All analyses for statistical significance were done in JMP (SAS). Tests for significance were done at an alpha level of α = 0.05.

Previous work has shown that low-frequency TFS provides crucial information for sound localization [28,29,32], leading us to hypothesize that stimuli in the Chimaera HP category would be showing a greater range of RMS errors, compared to stimuli in the Chimaera category. We thus used a one-tailed paired t-test between conditions in the Chimaera and Chimaera HP category to assess whether low-frequency TFS increased RMS errors of stationary localization. Further, previous research showed no difference in localization of stationary speech or noise sounds for NH listeners [13], leading us to compare RMS errors for the two stimuli in the Unprocessed category (OS, SMN) using a two-tailed t-test. To evaluate the impact of ENV on RMS errors, we used a two-tailed t-test to compare the performance for conditions which held the TFS constant, but varied in the ENV (OS-NC, SMN-SC). A total of four paired t-tests are needed to fully examine the effects of ENV and TFS in each experiment. With six levels of stimuli condition, which allows up to five pair comparisons, no correction is needed to address potential inflation of Type I error for multiple comparisons.

In the two main experiments, we investigated the impact of low-frequency TFS (present/absent) and envelope type (fluctuating/flat) on auditory motion perception. To understand how a participant’s ability to detect sound motion and their response bias was affected, we calculated senstivity (*d’*) and a response bias criterion (*c*) for each condition. Previous work indicated that removing access to low-frequency TFS is unlikely to increase sensitivity to sound motion detection [18,42], leading us to hypothesize that removing access to low-frequency TFS would reduce sensitivity to sound motion identification. As such, we tested the effect of low-frequency TFS on sensitivity to sound motion using a one-tailed t-test between conditions that differed only in their availability of low-frequency TFS, while keeping the envelope constant: SC-SC_hp and NC-NC_hp (see Table 1). Further, a fluctuating ENV provides more dynamic changes of interaural cues, compared to a flat ENV, which led us to predict that stimuli with a speech ENV showed better sound motion detection, compared to stimuli with an SMN ENV. To assess the impact of ENV on participants’ sensitivity to sound motion, we used a two-tailed t-test between conditions that differed in their envelope, while keeping the TFS constant: OS-NC, and SMN-SC, respectively (see Table 1).

*Low-frequency* TFS has previously been shown to degrade stationary lateralization and localization [30–33], and we were thus not interested in investigating its impact on response bias. However, we wanted to understand the relative impact of *full bandwidth* TFS and signal ENV on response bias, and thus used the four conditions with reliable signal ENV and TFS information (OS, SMN, SC, NC) for statistical testing. To assess the impact of TFS on response bias, we used a two-tailed t-test between conditions that differed in their TFS, while keeping the ENV constant (SMN-NC; OS-SC). To assess the impact of ENV on response bias, we used a two-tailed t-test between conditions that differed in their ENV, while keeping the TFS constant (SMN-SC; OS-NC; see Table 1).

## Results

In this study, we utilized chimaera-type stimuli to evaluate the usefulness of signal ENV and low-frequency TFS to auditory motion perception. We evaluated the sensitivity (*d’*) and response bias (*c*) of sound motion detection. For clarity of reading the results section, mean and standard error of the mean (sem) for each condition of the main Experiments I and II are reported in Table 1, which also lists percent correct speech understanding scores per condition.

### Stationary sound localization

**Fig 1:**
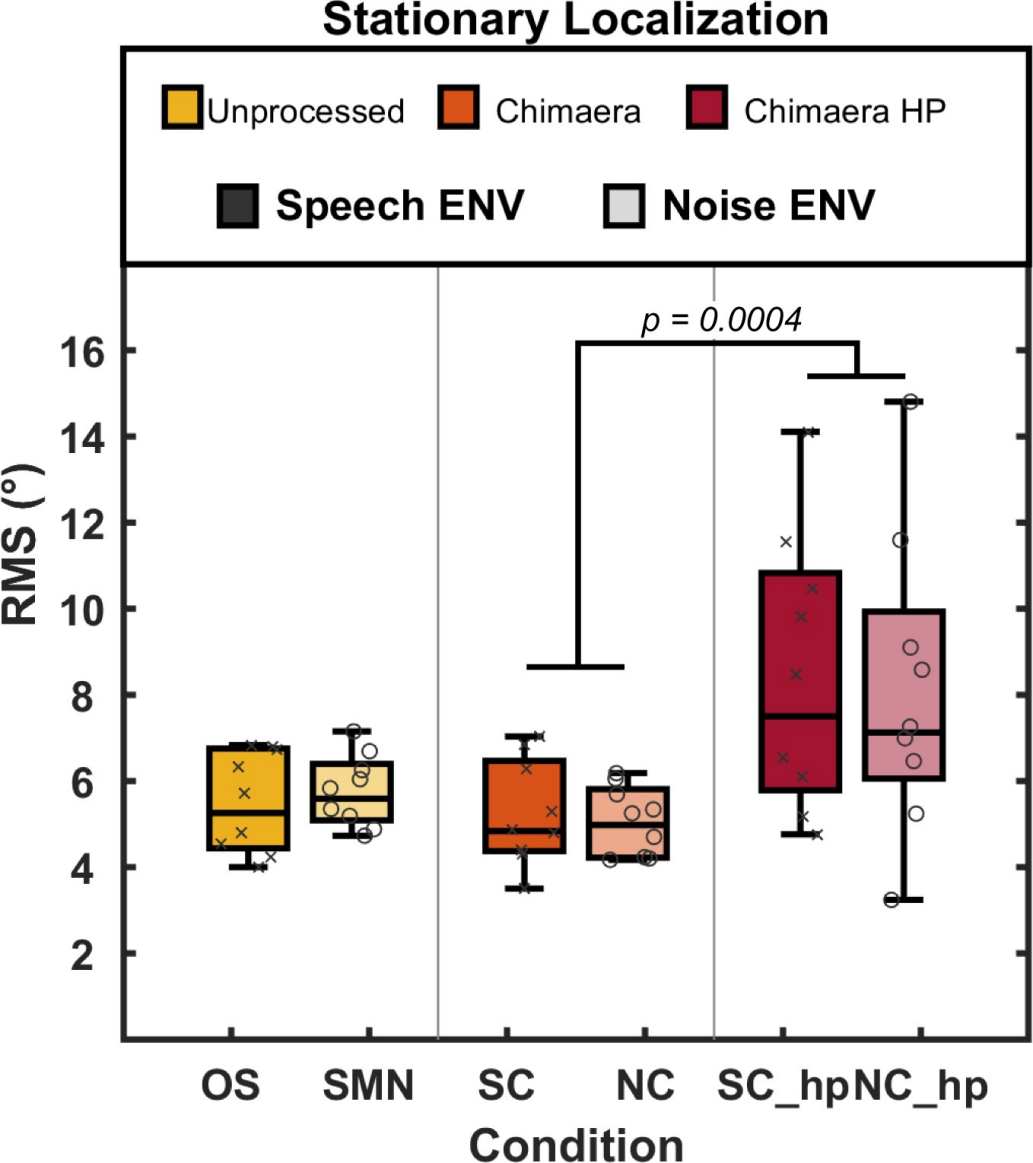
RMS errors for stationary sound localization. RMS errors (y-axis) collected during stationary sound localization testing across conditions (x-axis) are plotted as boxplots with group medians (black line) and individual data (individual markers) superimposed. Data are organized by stimulus category (color legend), and ENV type is indicated by color intensity (legend).

To understand how TFS and signal ENV impact stationary localization, we calculated RMS error as a measure of localization accuracy. Boxplots in Fig 1 show the RMS error (º; y-axis) for each stimulus condition (x-axis) with individual data points superimposed. RMS errors for individual participants ranged from 3.2° to 14.8° across the six conditions. We observed no significant differences between localization errors of stationary speech (OS) or SMN signals (*t*(14) = 0.50, *p* = 0.62), which corroborated previous findings that, unlike BiCI users, NH listeners do not localize speech signals more accurately than noise signals [13,36,37]. To assess whether removing low-frequency TFS increased listeners’ stationary localization errors, we compared RMS errors between categories that differed only in the availability of low-frequency TFS (Chimaera vs. Chimaera HP), and found a significantly larger RMS errors for stimuli in the Chimaera HP category (mean = 8.3°, ± s.e.m. = 0.75°), as compared to stimuli in the Chimaera category (mean = 5.18°, ± s.e.m. = 0.23°; *t*(20) = 3.98, *p* = 0.0004). Furthermore, signal ENV did not significantly impact stationary localization, independently of whether the TFS was speech (OS-NC; *t*(14) = 0.97, *p* = 0.34) or SMN (SMN-SC; *t*(14) = 1.08, *p* = 0.29).

### Sensitivity to sound motion

**Fig 2:**
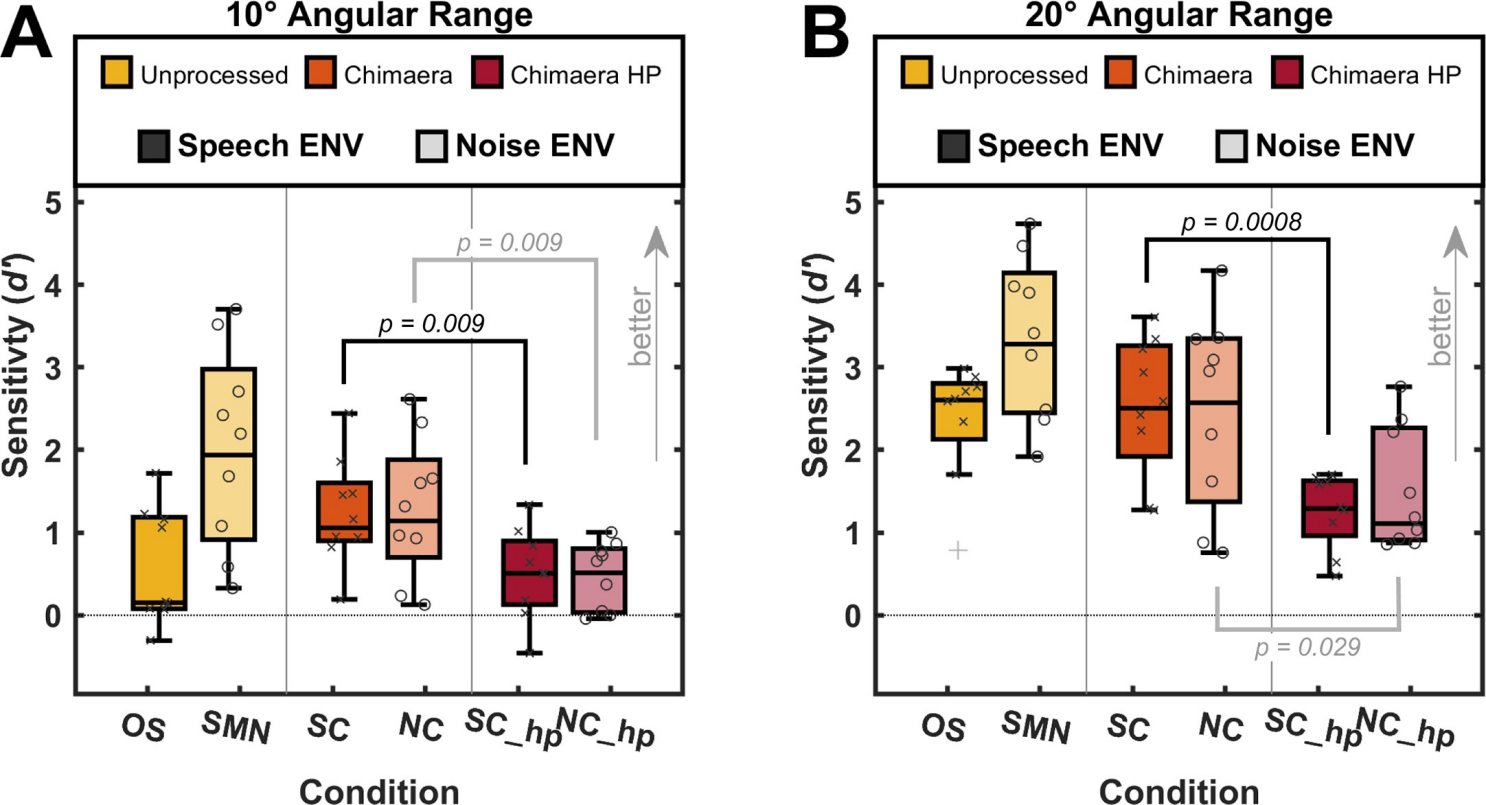
Sensitivity values for Experiment I and II. (A) Sensitivity values (*d’*, y-axis) for motion detection when sounds were stationary or moved 10° angular distance across conditions (x-axis). Plotted are boxplots with group medians (black line) and individual data (individual markers) superimposed. Data are organized by stimulus category (color legend), and ENV type is indicated by color intensity (legend). Plus-signs denote outliers. (B) same as (A) for motion detection when sounds were stationary or moved 20° angular distance.

In both main experiments, sounds were either stationary or moving. To understand how well listeners could distinguish between stationary and moving sounds, we calculated *d’* as a measure of sensitivity. Boxplots in Fig 2A illustrate listeners’ sensitivity in detecting sound motion when moving sounds traveled 10° angular distance (Experiment I; y-axis). Data are grouped by stimulus category (color legend) and plotted across conditions (x-axis) with individual data points superimposed. A greater *d’* value indicates better sensitivity for sound motion detection.

We investigated the impact of low-frequency TFS on sensitivity to sound motion by comparing sound motion detection sensitivity to chimaera stimuli with and without HP filtering within the two ENV types. When stimuli contained a fluctuating speech envelope (i.e., SC, SC_hp), removing low-frequency TFS significantly reduced listeners’ ability to distinguish between stationary and moving sounds (*t*(15) = -2.64, *p* = 0.009). A similar result was found when stimuli had a flat SMN ENV (i.e., NC, NC_hp; *t*(11) = -2.71, *p* = 0.009). Importantly, this significantly reduced sensitivity between fluctuating and flat ENV types to motion identification was also observed when moving stimuli traveled 20° angular distance (Fig 2B, Experiment II; fluctuating speech ENV: SC, SC_hp, *t*(12) = -4.03, *p* = 0.0008; flat SMN ENV: NC, NC_hp, *t*(13) = -2.06, *p* = 0.029).

Next, we investigated the impact of ENV on sensitivity to sound motion, by comparing pairs of chimaeras with the same TFS, but different types of ENV. When stimuli were either stationary or moved 10° angular distance (Experiment I, Fig 2A) and had SMN in their TFS (i.e., SMN, SC), we found no significant effect of ENV type on listeners’ ability to distinguish between stationary and moving sounds (*t*(12) = 1.68, *p* = 0.11). The same result was found when stimuli had speech as their TFS (OS, NC; *t*(15) = -2.0, *p* = 0.062). Further, increasing the angular distance of sound motion to 20° (Experiment II; Fig 2B) showed no significant impact of ENV on sound motion detection (SMN TFS: *t*(15) = -1.93, *p* = 0.071; speech TFS: *t*(13) = -0.23, *p* = 0.81).

### Response bias to sound motion

**Fig 3:**
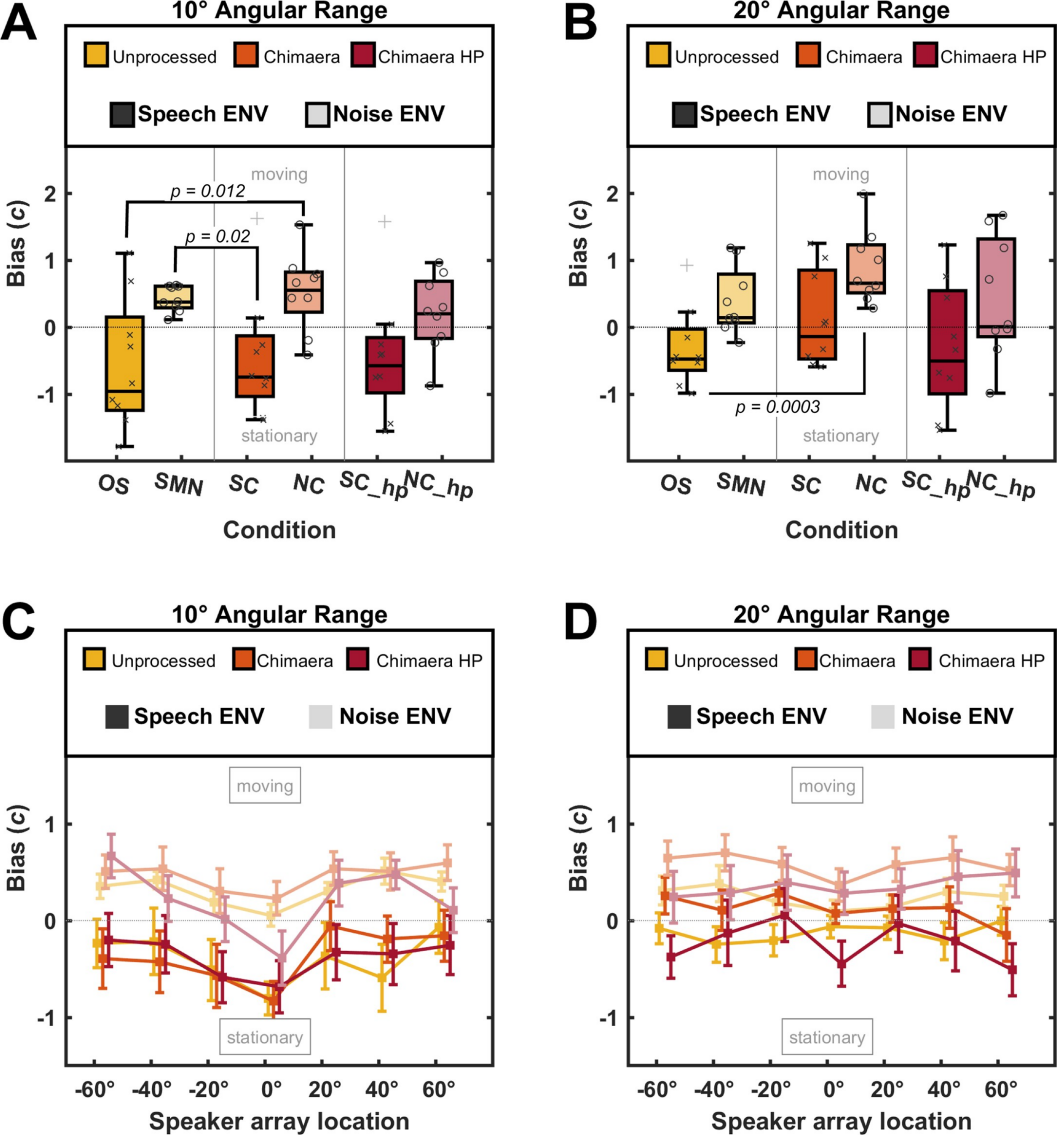
Bias values for Experiment I and II. (A) Bias criteria (*c*, y-axis) for motion detection when sounds were stationary or moved a 10° angular distance across conditions (x-axis). Plotted are boxplots that mark group medians (black line) and individual data (individual markers) superimposed. Data are organized by stimulus category (color legend), and ENV type is indicated by color intensity (legend). Plus-signs denote outliers. Positive values indicate a bias to judge sounds as moving, while negative values indicate a bias to judge sounds as stationary. (B) same as (A) for motion detection when sounds were stationary or moved a 20° angular distance. (C) Bias criteria (*c*, y-axis) from (A) as a function of sound origin location (x-axis). Mean (± sem) critera (square) are indicated for each category (colored legend), and ENV type is indicated by color intensity (legend). (D) same as (C) for equivalent data in (B).

Evaluating the listener’s sensitivity to sound motion for different stimuli helped us understand how well they could detect sound motion. Beyond identification accuracy, we were interested in learning whether TFS or ENV induced a response bias in judging sounds as moving or stationary. Fig 3A plots the response bias criterion (*c*, y-axis) for individuals and group data across the six conditions (x-axis), for sound motions of 10° angular distance (Experiment I). A bias criterion of 0, indicates no bias to either sound motion; bias criteria > 0 or < 0 indicate a bias towards judging sounds as moving, or stationary, respectively.

We investigated the impact of TFS on response bias by analyzing conditions which held the ENV constant, but varied in their TFS. When stimuli contained a fluctuating ENV (OS, SC), the type of TFS did not significantly affect listeners’ judgement of sound motion (*t*(15) = 0.23, *p* = 0.82). Similarly, when stimuli had a flat ENV (SMN, NC), TFS did not affect response bias (*t*(9) = -0.52, *p* = 0.60). Further, increasing the angular distance of sound motion to 20° (Fig 3B, Experiment II) also showed no effect of TFS on response bias (OS-SC: *t*(15) = 1.47, *p* = 0.16; SMN-NC: *t*(15) = -2.09, *p* = 0.052).

We investigated the impact of ENV on response bias by analyzing conditions which held the TFS constant, but varied in their ENV. When stimuli contained speech in their TFS (OS, NC), we found that a fluctuating ENV showed a significantly lower bias criterion compared to a flat ENV (*t*(12) = -2.88, *p* = 0.012). Additionally, when stimuli contained SMN in their TFS (SC, SMN), a similar result was discovered: stimuli with a fluctuating ENV had significantly lower response bias criteria than stimuli with a flat ENV (*t*(8) = 2.78, *p* = 0.02). When listeners judged sound motion detection during Experiment II, where angular distance of sound motion was 20º (Fig 3B), a significant difference in response bias remained for stimuli that had speech in their TFS (*t*(15) = -4.56, *p* = 0.0003), but was not observed for stimuli with SMN in their TFS (*t*(14) = 0.84, *p* = 0.41).

Fig 3A and 3B illustrate that signal ENV, but not TFS, significantly impacts sound motion bias, indicating that sounds with a fluctuating ENV are perceived more stationary-like compared to stimuli with a flat ENV. We questioned whether this trend is true independent of where the sound occurred. A plethora of previous studies has suggested remarkable resolution for stationary sound localization in the foveal region of the frontal hemifield [8,10,21,43]. This would suggest that response bias is more strongly impacted by sound presentations at peripheral locations, where spatial resolution is increasingly coarse [8,10,21,43], as reduced resolution might bias listeners to judge sounds as stationary or moving. Fig 3C and 3D plot the mean (± sem) response bias (y-axis) at different locations along the horizontal loudspeaker array (y-axis) for sound sources moving an angular distance of 10° or 20°, respectively. Contrary to our expectation, response biases in Experiment I (Fig 3C) form a v-shaped pattern as a function of onset location, showing greatest response biases (smallest bias criteria) toward identifying sounds as stationary at 0° azimuth. This pattern can be observed independently of whether stimuli had a fluctuating (strong color) or flat (pastel color) signal ENV. By contrast, when moving sounds traversed a 20° angular distance (Experiment II; Fig 3D), the v-shaped pattern largely disappeared, and response criteria were mostly the same within separate conditions (colors and shading) across different locations of the loudspeaker array.

## Discussion

The auditory cues that help NH listeners detect, identify, locate, and extract information from moving sounds are not well understood. This is true even to a greater extent for listeners with hearing loss, for whom there is only limited research available (e.g. [18,24]). Recently, Moua and colleagues ([18]) investigated how parameters such as stimulus duration and angular velocity impact auditory motion perception in NH and BiCI listeners. Their findings show that BiCI listeners generally performed worse than NH listeners in identifying sound motion. CI processing removes TFS, but retains the signal ENV of the original acoustic input to amplitude-modulate an electric pulse train. While this processing strategy yields good speech perception in quiet for BiCI users [3,25], their sound localization performance is poor, as indicated by large RMS errors averaging 20° to 30° (e.g. [4,13]). Low-frequency TFS below 2.5 kHz, which BiCI users do not have access to, delivers ITDs crucial for lateralization and localization of stationary sounds in NH listeners [8,9,28–32]. The present results confirmed our prediction that removing low-frequency TFS would reduce the accuracy of stationary sound localization (Fig 1) and the ability to detect moving sounds (Fig 2). These results suggest that the performance gap between NH and BiCI listeners in identifying sound motion reported by Moua and colleagues, and accurately localizing sound sources, may be due to the lack of access to low-frequency TFS, which is known to convey important auditory cues for localizing stationary sounds [2,7,14,25].

The present study aimed to understand the individual contributions of low-frequency TFS and ENV in the acoustic signal to sound motion detection among NH listeners. When sounds are in motion along the horizontal plane, binaural cues such as ITDs and ILDs vary rapidly during a very short time window. It is possible that having access to precisely mapped ITDs and ILDs is even more critical for detecting auditory motion than for perceiving stationary sound source locations. Utilizing chimaera stimuli, which had the ENV of one signal (e.g., speech) and TFS of another signal (e.g., SMN), we tested NH listeners’ perception of sound motion for stimuli with varying ENV and TFS for angular motion. Our results indicate that signal ENV did not affect the sensitivity to sound motion detection among NH listeners. However, contrary to our expectations, we found that chimaera stimuli with a fluctuating speech ENV biased listeners to perceive sounds as stationary-like, compared to chimaera stimuli with a flat noise ENV (Fig 3). The perceptual biases were stronger when the angular distance of sound motion was 10° (Experiment I) than when it was 20° (Experiment II).

Collectively, these results suggest two conclusions. First, low-frequency TFS impacts auditory motion perception, in that not having access to this cue reduces the ability to distinguish between stationary and moving sounds. This phenomenon seems to persist in angular distances up to at least 20°. Second, listeners are biased toward reporting a more stationary percept when the signal ENV is fluctuating, such as from speech signals. Conversely, NH listeners are slightly biased towards reporting sounds as moving when the ENV is flat, such as for SMN signals. This effect, however, seems to be restricted to sounds that move a smaller distance, where distinguishing sound motion is in general more challenging. This suggests that as distinguishing between stationary and moving sounds becomes easier, because the moving sound traverses a large angular distance, ENV cues become less restrictive.

Previous research has suggested that the binaural system can follow interaural level difference cues more efficiently than ITD cues [44,45], and that a dynamic ILD cue may be more salient for motion velocity perception, than a dynamic ITD cue [46]. While TFS provides access to ITD, we speculate that ENV may facilitate sound motion detection on the basis of dynamically changing ILD cues. Specifically, we posit that listeners may have been tracking the changes of the signal envelope as a sound traversed across an array of spatial locations. When the signal envelope changes in time *and* space, as it does for signals with unpredictable fluctuating ENVs such as speech, this task becomes challenging. This is because the difference between the level estimate from ILD cues at the current location and the level estimate from ILD cues at the previous location may not be due to a change in location, but due to the change in level of the signal itself. As a result, the sound motion of signals with quasi randomly fluctuating ENVs, such as speech signals, would likely be more biased to be perceived as stationary. By contrast, signals with flat ENVs, for which a listener would be comparing approximately equal energy levels from one spatial location to the next, might be perceived with smaller biases. In other words, accurate motion detection would be hindered by signals with a fluctuating ENV, but facilitated by signals with a flat ENV.

Creating a virtual sound source that is perceived to be moving smoothly can be challenging for some acoustic stimuli. For example, previous work using VBAP showed that listeners could detect spectral changes of relatively long (several seconds) continuous pink noise that moved 360° around the listener’s head, at an angular velocity of 100°/s [47]. We addressed this challenge by creating individual inverse filters to “flatten” the output frequency responses of the loudspeakers (see Methods). Further, the stimuli in our experiment were comparatively short (~ 500 ms) and moved only 10° or 20° in angular distance at an angular velocity of ~ 20°/s to 40°/s. Informally, participants reported perceiving smoothly moving sounds during the experiments.

For the angular sensitivity of stationary sounds, the minimum audible angle (MAA) has been quantified in some studies as the smallest angular separation that can be detected between sound sources –the key parameter being that the two sources are presented successively at two locations. MAA is smallest in the frontal foveal hemifield, around 1° [25,43,48–51]. This led us to predict that perhaps sound motion detection would be *least* biased at frontal locations. Surprisingly, however, we found that listeners showed a strong bias towards judging sounds as stationary in frontal locations, particularly at 0° azimuth (Fig 3C). This pattern was observed ubiquitously across all stimulus types, though it largely disappeared when sounds moved 20º angular distance. We thus interpret our findings to suggest that increasing angular distance provides the listener with a greater range of changes in acoustic ITD and ILD cues across spatial locations, thereby increasing the listener’s sensitivity to sound motion detection, and reducing the response bias.

The most unbiased responses at the frontal (0° azimuth loudspeaker) location were seen with SMN stimuli (see Fig 3C). SMN is similar to a broadband noise, which is a common stimulus for stationary localization and testing of MAAs [8,10,43,48,51]. Considering that the MAA is smallest at foveal locations, it is conceivable that there would be little or no bias for a broadband noise. Interestingly, the SMN is the only type of stimulus used here that has no speech intelligibility; all remaining stimuli contained either speech TFS or a speech ENV, and provided between 6% to 100% speech understanding in our task (Table 1). While we found no difference in the ability of NH listeners to localize speech (OS) or noise (SMN) stimuli (Fig 1) (cf. [13,36,37]), it is possible that response bias of sound motion is impacted by the degree of speech intelligibility in each condition. Fig 2A shows that NH listeners’ ability to distinguish stationary from moving sounds was low for speech stimuli in the OS condition, with a median *d’* of 0.21 –the lowest sensitivity across conditions tested here. This indicates that listeners’ ability to distinguish moving speech from stationary speech is very poor. Further, the response bias for stimuli in the OS condition were perceived as more stationary, compared to SMN stimuli (Fig 3A), suggesting that unprocessed speech stimuli may be biased to be judged as more stationary.

This hypothesis is supported upon close inspection of the response bias and speech perception scores listed in Table 1. The conditions SMN and OS create the lower and upper limits of correct speech perception, with average speech perception scores of 0% and 99.8%, respectively. The OS condition has the smallest response bias (median: -0.87), indicating that these stimuli were judged as more stationary compared to the SMN conditions, where the response bias is larger (median: 0.38; Fig 3). Furthermore, the SC and NC conditions follow the same pattern. Speech perception scores for the SC condition average about 90% correct, and the response bias is smaller (median: -0.72) than that of the the NC condition (median: 0.66; Fig 3), for which speech perception averages about 44% correct. As removing low-frequency TFS affected the ability to localize stimuli and distinguish their sound motion, these conditions (SC_hp, NC_hp) should not be considered for this comparison. These patterns suggest that as the speech perception scores increase, the response bias decreases. This could indicate that as listeners understood more of the presented stimulus’ content, they were also more likely to perceive it as stationary. However, given that both the SMN and NC conditions presented the listeners with a flat ENV, while the OS and SC conditions presented the listeners with a fluctuating ENV, we are unable to determine whether this effect is truly due to the amount of speech perception tied to each condition, or merely an effect or signal ENV; there may also be an interaction between these two components.

Using a dual-task, we observed a potential trade-off between sensitivity to auditory motion and speech understanding. It is unclear whether this observation is a spurious byproduct of our data. The findings from this study open up the question of whether sound motion detection is modulated by auditory attention. That is, bias toward a stationary percept may be due to listeners failing to process auditory cues in the TFS and ENV when they hear a speech token that is highly intelligible. Indeed, future work is needed to explore the role of auditory attention in auditory motion beyond auditory cues in the TFS and ENV.

## Conclusions

This study investigated the usefulness of low-frequency TFS and signal ENV for sound motion identification in NH listeners. We found that

1. removing low-frequency TFS reduces listeners’ sensitivity to sound motion detection, indicating that TFS is an important cue for auditory motion perception.
2. the impact of low-frequency TFS is consistent up to at least 20° angular displacement.
3. signal ENV affects response bias of categorical sound motion identification, indicating that a fluctuating (speech) ENV biases listeners to perceive a sound as more stationary compared to a flat (noise) ENV.
4. response bias is most strongly affected at locations in the frontal hemifield, indicating that sound motion detection is less biased, or more accurate, at peripheral locations.
5. the impact of signal ENV on response bias is stronger when angular distances are smaller, and decreases as angular distance increases and motion detection becomes easier.

## Supporting information

S1 Fig Categorization via k-means clustering of sound motion tracking data. Figure plots the raw tracked angular distance (y-axis) per condition (rows) for each participant (column) in Experiment I. Data points with black edge color plot trials in which the stimulus was moving at 10º angular distance, while data points with edge color matching the condition color plot trials in which the stimulus was stationary. Red line indicates threshold at which k-means clustering separated the data cloud.
(TIF)

S1 Appendix Minimal anonymized dataset to replicate the findings of this study. An excel work book with sheets for the different data methods utilized for analysis of stationary localization, sound motion sensitivity, and sound motion response bias is provided.
(XLSX)
