# Supplementary figures and images for: The impact of temporal fine structure and signal envelope on auditory motion perception
Source: PLoS One. 2020 Aug 21;15(8):e0238125. doi: 10.1371/journal.pone.0238125 (PMC7446836; doi:10.1371/journal.pone.0238125)

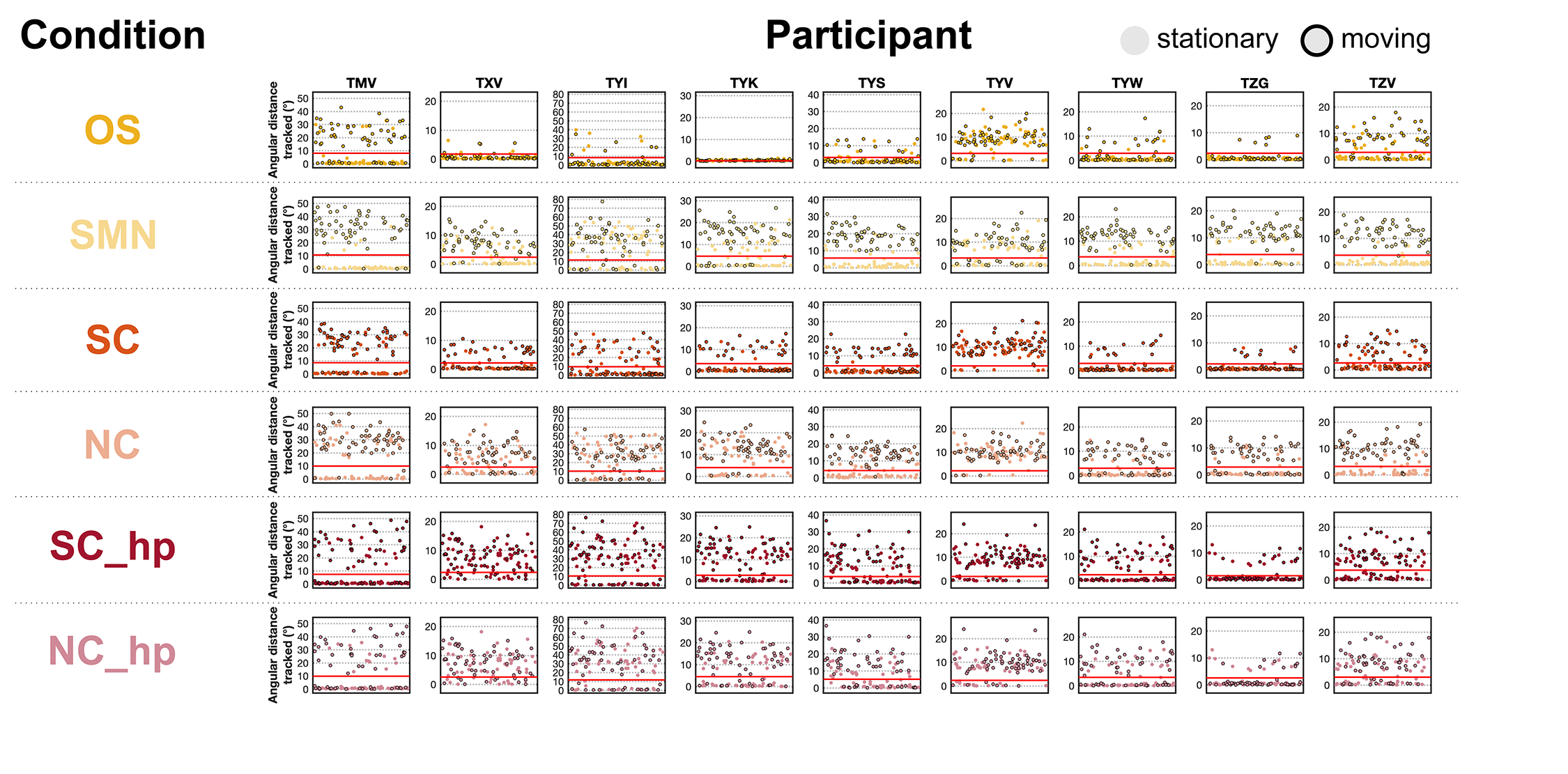

Supplement: S1 Fig — Figure plots the raw tracked angular distance (y-axis) per condition (rows) for each participant (column) in Experiment I. Data points with black edge color plot trials in which the stimulus was moving at 10º angular distance, while data points with edge color matching the condition color plot trials in which the stimulus was stationary. Red line indicates threshold at which k-means clustering separated the data cloud. (TIF) [file pone.0238125.s001.tif]
